# Atlas of Signaling for Interpretation of Microarray Experiments

**DOI:** 10.1371/journal.pone.0009256

**Published:** 2010-02-17

**Authors:** Ekaterina Kotelnikova, Natalia Ivanikova, Andrey Kalinin, Anton Yuryev, Nikolai Daraselia

**Affiliations:** Ariadne Genomics, Inc., Rockville, Maryland, United States of America; Georgia Institute of Technology, United States of America

## Abstract

Microarray-based expression profiling of living systems is a quick and inexpensive method to obtain insights into the nature of various diseases and phenotypes. A typical microarray profile can yield hundreds or even thousands of differentially expressed genes and finding biologically plausible themes or regulatory mechanisms underlying these changes is a non-trivial and daunting task. We describe a novel approach for systems-level interpretation of microarray expression data using a manually constructed “overview” pathway depicting the main cellular signaling channels (Atlas of Signaling). Currently, the developed pathway focuses on signal transduction from surface receptors to transcription factors and further transcriptional regulation of cellular “workhorse” proteins. We show how the constructed Atlas of Signaling in combination with an enrichment analysis algorithm allows quick identification and visualization of the main signaling cascades and cellular processes affected in a gene expression profiling experiment. We validate our approach using several publicly available gene expression datasets.

## Introduction

Microarray-based expression profiling of living systems is a quick and inexpensive method to obtain insights into the nature of various diseases and phenotypes. It is also a great way of studying the functions of individual proteins or drugs by looking at the affected targets after system distrurbances or genetic modifications (siRNA, knock-outs, gene over-expression, etc).

The greatest challenge of microarray-based expression profiling is interpreting the obtained results. In a typical microarray experiment, mRNA profiles are generated for thousands of genes on a chip from a collection of samples derived from studied experimental conditions. Thus, the difficulty is finding an underlying biological theme or specific mechanisms hidden behind the expression profiles. Many of the genes changed in an experiment may fall outside the area of expertise of an individual researcher. A common approach has always been focusing on a handful of most highly changed probes. The main limitation of this approach is a risk to miss small, but concerted changes in a group of functionally related genes.

The recent advancement in interpreting the microarray data is development of the gene set enrichment analysis (GSEA) [1]–a statistically robust algorithm which compares the entire differential expression profile against biologically meaningful gene sets, defined by prior knowledge (e.g. pathways, cellular processes, etc). The goal of the GSEA is to determine whether all members of each gene set tend to be synchronously changed in a microarray experiment. As a result, the microarray experiment is projected on a much smaller list of statistically significantly changed gene sets which can summarize the observed expressional changes on a gene-systems level. A drawback of focusing only on highly differentially expressed genes lies in the fact that signaling proteins participating in the observed cellular response might not be changed on the level of expression even though corresponding pathways are activated or inhibited.

In this paper, we present a novel approach for analysis of differential gene expression profiles aimed at identification of key protein regulators and pathways involved in the differential response. The major ideas of our approach are:

- Utilizing a gene expression regulatory network built using facts extracted from literature to generate a comprehensive collection of gene sets, each representing immediate downstream neighbors (sub-networks) of every individual protein in the network.
- Grouping proteins into functionally coherent groups (either by protein families performing similar functions or by participation in common cellular processes) and connecting these groups by well-established biological regulatory links into a single overview pathway (Atlas of Signaling) depicting main cellular signaling channels.
- Interpreting differential gene expression by “projecting” sub-networks significantly enriched with differentially expressed genes onto the Atlas of Signaling in order to identify key regulatory proteins and pathways involved in the differential response.

Using publicly available gene expression datasets, we demonstrate that this approach can successfully identify main signaling cascades involved in the regulation of the cellular response.

## Methods

All the analyses described in this paper have been performed using PathwayStudio® software version 6.2. PathwayStudio is a commercial product for pathway analysis which contains a comprehensive database of protein–protein relationships extracted from literature using MedScan®–a fully automated biomedical information extraction engine.

### An Overview of the Atlas of Signaling

The principal components of the Atlas of Signaling are protein groups (classes) representing either protein families or molecular-level cellular processes. Conceptually, we distinguish 5 sub-categories of proteins: ligands, receptors, signaling proteins, transcription factors and “workhourse” proteins (Table 1).

**Table 1:** The main categories and subcategories of protein groups with annotation statistics^a^.

Category	Sub-categories	Number of groups/classes	Number of proteins
Molecular function	Ligands	42	444
	Receptors	65	932
	Signaling proteins	157	744
	Transcription factors	94	1,841
Cellular Process	Biochemical processes	41	1,579
	Metabolite/ion transport	42	1,056
	Structural processes	63	3,821

a The complete list of protein classes can be found in supporting File S1.

Workhorse proteins are grouped into cellular processes. We thought to develop a minimal set of ubiquitous tissue-independent molecular events intrinsic to normal physiology of a eukaryotic cell, e.g. actin cytoskeleton assembly, DNA replication, or translation. More complex structural processes, such as mitosis, apoptosis or vesicular transport can be represented in terms of these elementary processes. For instance, the molecular events of mitosis include chromatin condensation, spindle assembly, centrosome separation, kinetochore assembly etc. The higher-level structural process can be represented as a chain of elementary events, each performed by a limited set of proteins-executors. The same pertains to the majority of tissue-specific processes. For example, the process “neurotransmitter secretion” describes a neuron-specific version of secretory vesicle exocytosis. Proteins of the SNARE complex and clathrin cage proteins comprise the group of executors responsible for membrane budding and fusion during various exocytosis events in different cell types. We assigned only direct executors (“workhorse proteins”) or their direct specific regulators to our minimal set of cellular processes: biochemical pathways include only biochemical enzymes, transport processes include only transporters, and structural processes include structural proteins responsible for physical integrity of a cell or its “molecular machinery” and their direct specific regulators (e.g. regulatory cytoskeleton- or microtubule-associated proteins). For instance, the “microtubule sliding” process contains tubulins, kinesins, dyneins and microtubule-asociated proteins. Cellular processes also include biochemical and transport processes that contain major cellular biochemical pathways and metabolite/ion transport processes respectively.

Ligands, receptors, signaling proteins, and transcription factors are grouped into protein families based on sequence and functional similarity. The complete list of protein groups can be found in the supporting File S1.

**Figure 1:**
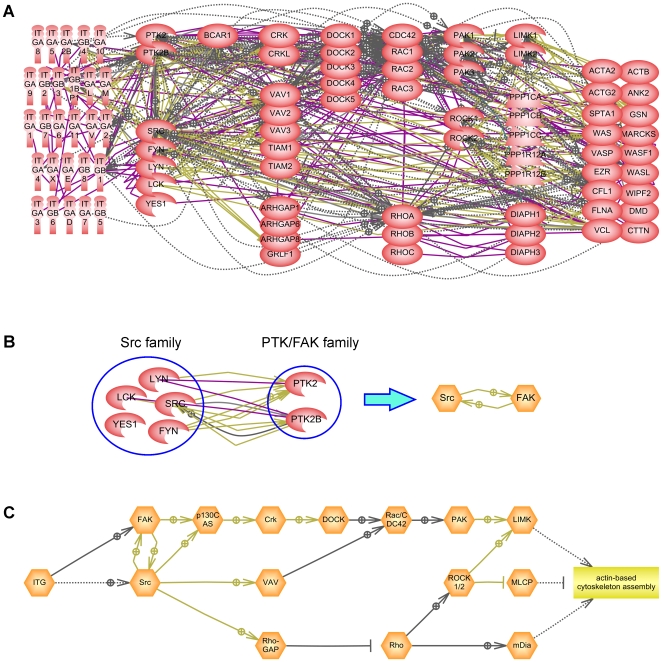
Reduction of a pathway complexity using Atlas of Signaling. (A) Signal transduction pathway from integrin receptors to actin cytoskeleton built from individual proteins (red shapes) using relationships found in ResNet 6 database. Entrez official gene symbols are used for protein names. (B) Example of how a relation between two groups (functional classes) is formed in the Atlas of Signaling. (C) Same pathway as in (A) built with ontological groups: functional classes (orange hexagons) and cell process “actin-based cytoskeleton assembly” (yellow rectangle). The description of relationships between entities on the pathways is provided in the legend to Table 2.

**Figure 2:**
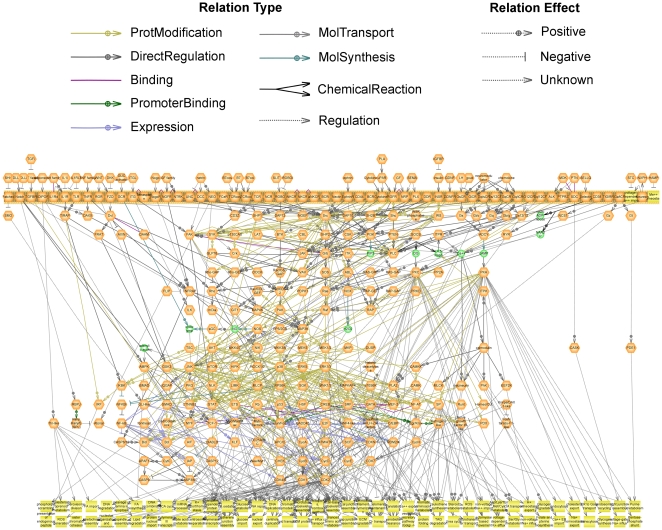
Atlas of Signaling. The major known signaling cascades were combined in one pathway diagram to provide “birds-eye” view of cellular signaling. Signaling functional groups are shown as orange hexagons; secondary messengers/small molecules–as green circles, and cellular processes–as yellow rectangles.

We have connected protein groups by regulatory relationships that are used to describe the signaling information flow inside the cell. The relationships between protein classes are based on well-established relationships between individual proteins from the two classes (Figure 1) described in literature. There are several types of protein relationships that were originally introduced in ResNet database for Pathway Studio [2], [3]. The complete list of relationship types and their statistics on the Atlas of Signaling is shown in Table 2. All relationships between functional groups can be visualized in Pathway Studio as one big pathway that we call the “Atlas of Signaling” (Figure 2). The Atlas is focused on the transduction of signals from extracellular space through receptors and regulatory cascades to transcription factors and further regulation of expression of workhorse proteins. Extracellular ligands are on the top of the map followed by the row of plasma membrane-localized receptors. Cell processes are positioned at the bottom, and signaling proteins and transcription factors connect upper and lower parts of the map. The map contains 381 protein classes and 861 relations and can be considered a “scaffold” map of the main signal transduction pathways in a cell. The full list of relationships in the Atlas of Signaling is provided in the supporting File S2.

**Table 2:** Relation types on the Atlas of Signaling.

Relation type	Description	Count
ProtModification	Protein members of the regulator class phosphorylate or otherwise modify proteins in the target class	243
DirectRegulation	Protein members of one class bind and regulate proteins in another class	160
Binding	Protein members of one class bind proteins in another class	18
PromoterBinding	Protein members of one class bind promoters of genes encoding proteins in another class	8
Expression	Protein members of one class regulate expression of proteins in another class	28
MolTransport	Protein members of one class regulate export, import or release of proteins in another class	6
MolSynthesis	Protein members of one class regulate level of proteins in another class or level of a small molecule/metabolite	12
ChemicalReaction	Protein members of a class synthesize small molecule(s)/metabolite(s)	2
Regulation	Protein members of one class indirectly regulate proteins in another class	384

### Protein Expression Sub-Networks as Gene Sets

**Figure 3:**
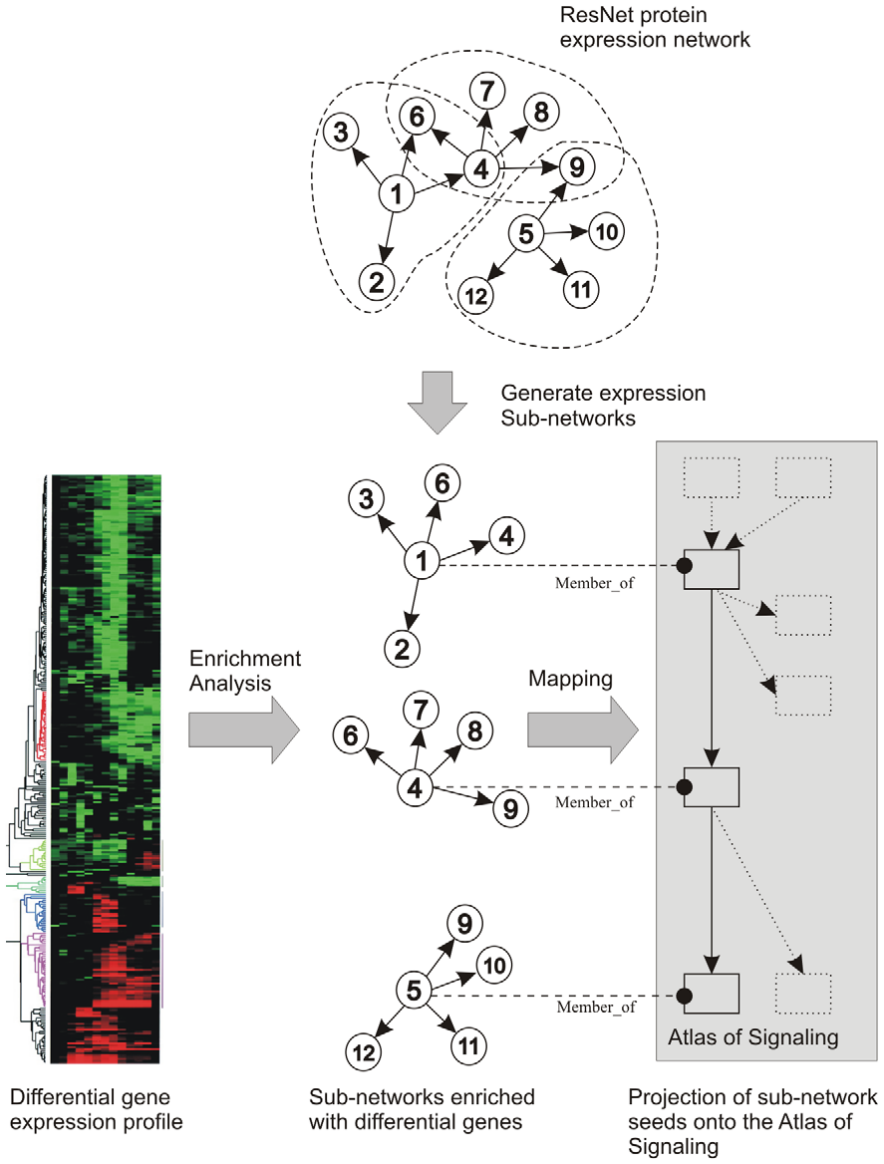
An overview of enrichment analysis and mapping onto the Atlas of Signaling. The ResNet gene expression regulatory network is split into individual sub-networks around each protein. Each sub-network contains immediate downstream neighbors of an individual protein in the network. In the depicted exemplary network, there are three sub-networks - build around “seed” proteins 1, 4 and 5; they are surrounded by dotted lines. The projection to the Atlas of Signaling is done by selecting the functional groups on the Atlas containing the seed proteins of sub-networks which were found to be statistically significant in the enrichment analysis. The three selected groups (rectangles) and relationships between them on the Atlas are shown in solid lines. The remaining (un-involved) groups and relationships are shown by dotted lines.

One of the key ideas behind our gene expression interpretation approach is utilization of a gene expression regulatory network built from facts extracted from literature. The network is used to generate a comprehensive collection of gene sets, each representing immediate downstream neighbors of each individual protein in the network (Figure 3). We call a “center” protein of such sub-network the “seed” protein and assume that if the downstream expression targets of the seed protein are enriched with differentially expressed genes (i.e. the sub-network is found to be statistically significant in enrichment analysis) then the seed protein is one of the key regulators of the observed differential response. Since sub-networks are constructed from all the proteins in the entire expression network, including ligands, receptors, signaling proteins and transcription factors, the seed proteins of statistically significant sub-networks presumably constitute the components of a regulatory network involved in the modulation of the observed differential response.

The gene expression regulation relationships are extracted from PubMed abstracts and full-text papers using MedScan technology [4], [5]. They describe involvement of various proteins: ligands, receptors, signaling proteins and transcription factors in regulation of protein expression. The complete gene expression regulatory network was extracted from entire 2007 PubMed and the content of 61 freely available full-text journals and contains 163,945 unique relationships. As noted, the extracted relationships represent not only direct regulation of expression by transcription factors, but also indirect relationships (e.g. “protein A regulates expression of protein B”, and similar statements).

In addition to expression sub-networks, the separate collection of gene sets was constructed from the members of all the functional groups themselves to capture the concerted changes in expression among the members of the groups: ligands, receptors, signaling groups and transcription factors, as well as cellular processes “groups”–biochemical pathways, transport processes and structural processes.

### Enrichment Analysis of Differential Gene Expression Using Expression Sub-Networks and the Atlas of Signaling

The following steps describe our approach for interpreting differential gene expression in the context of the Atlas of Signaling

1. Differential gene expression between samples of interest is calculated using T-test.
2. The enrichment analysis is performed for normalized log ratio differential samples using Mann-Whitney test [6] against the collection of gene sets generated as described above. All gene sets with a p-value <0.05 are considered significant and are selected for further analysis.
3. Significant gene sets are mapped to the Atlas of Signaling pathway as follows:
  - Gene sets corresponding to the cellular processes and functional groups are directly selected on the “Atlas of Signaling” pathway
  - Individual signaling/regulatory proteins representing the seeds of gene sets are first mapped to corresponding functional groups by containment, which are then selected on the “Atlas of Signaling” pathway.

All unmapped functional groups and cellular processes not selected by this mapping procedure are then removed from the Atlas of Signaling. The resulting “partial” pathway constitutes the “interpretation model” for a specific differential expression profile; it contains classes of proteins whose members or “expression targets” have revealed statistically significant changes in the differential expression profile. Figure 3 illustrates the procedure of sub-network generation and mapping the seeds of significant sub-networks onto the Atlas of Signaling.

## Results and Discussion

We have validated our approach by interpreting several publicly available expression microarray experiments using the Atlas of Signaling pathway. Since current focus of the Atlas is signal transduction from surface receptors to transcription factors, we have chosen experiments that measure response of normal human tissues to different hormones.

We first analyzed GDS1036 experiment from NCBI GEO that profiles gene expression in microglial cells in response to 200 u/ml interferon-gamma (IFN-gamma). IFN-gamma is a soluble cytokine and the only member of the type II class of interferons. In contrast to interferon-alpha and interferon-beta, which can be expressed by all cells, IFN-gamma is secreted only by T-lymphocytes, dendritic cells and NK cells. Produced by lymphocytes activated by specific antigens or mitogens, IFN-gamma, in addition to having antiviral activity, has important immunoregulatory functions. It is a potent activator of macrophages, it has anti-proliferative effects on transformed cells, and it can potentiate the antiviral and antitumor effects of the type I interferons.

**Figure 4:**
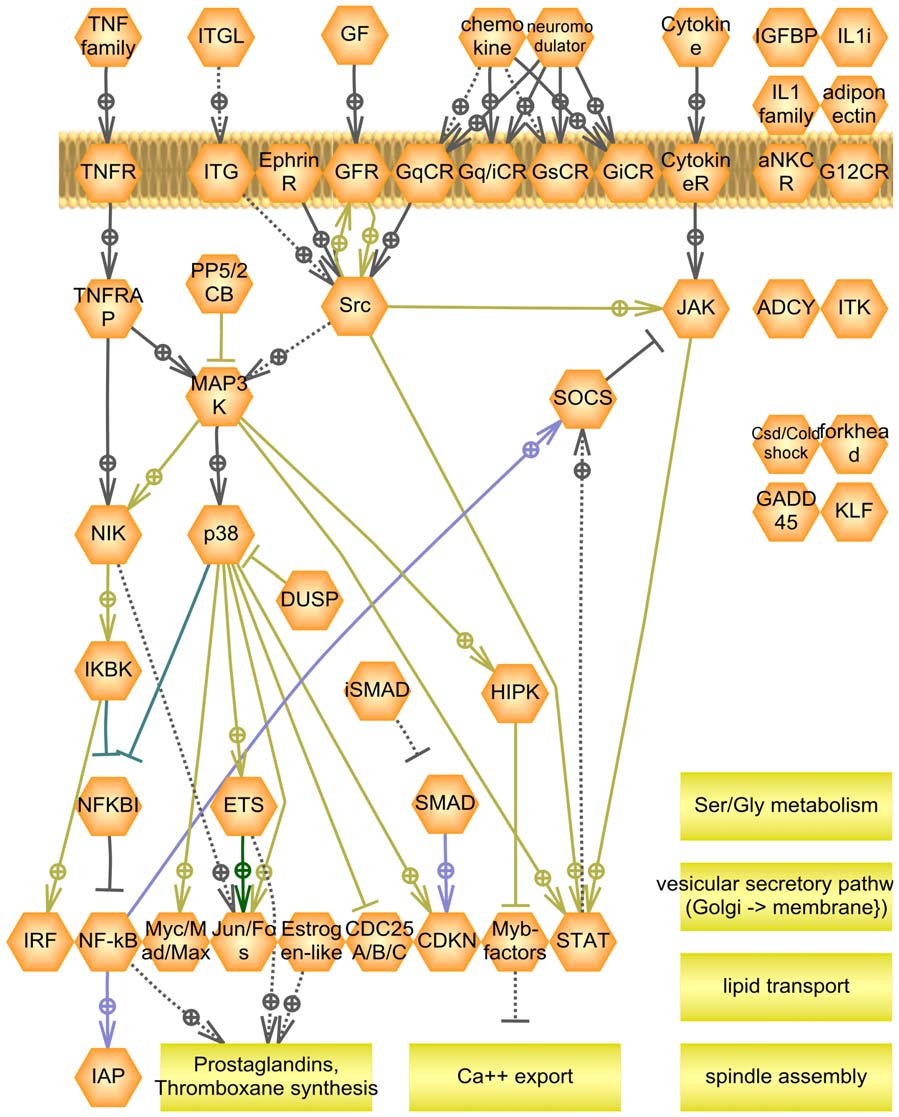
Projection of groups involved in IFN-gamma response in microglial cells onto Atlas of Signaling. The protein groups found by projection of the results of enrichment analysis of GEO dataset GDS1036 were connected using existing relations in Atlas of Signaling. Group shapes are as described in legend to Figure 2.

The imported data set contained three time points: 1, 6, and 24 hours of IFN-gamma exposure. Data for each time point were generated using microglial cell isolated from four different brain specimens. Analysis of differential expression between untreated samples and samples after 6 or 24 hours of exposure returned very similar lists of differentially expressed genes. Therefore, for further analysis, time points 6 and 24 hours were combined and compared to corresponding untreated control samples. The differential gene expression was calculated, followed by enrichment analysis using Mann-Whitney test [6] against “expression subnetwork” gene sets as described above. The significant sub-networks (p<0.05) were mapped on the Atlas of Signaling and the resulting pathway is shown on Figure 4. The majority of mapped functional classes on the Atlas of Signaling formed a well-connected sub-network with principal components being the classical JAK-STAT pathway and interferon-response factors (IRFs) that have been shown to be downstream of IFN-gamma in numerous publications [7], [8]. In addition, such cell processes as prostaglandin synthesis, spindle assembly, lipid transport, and Ser/Gly metabolism were also affected by IFN-gamma treatment in microglial cells. Down-regulation of prostanoid production by IFN-gamma has been shown previously [9]. Activation of spindle assembly and Ser/Gly metabolism are indicative of cell proliferation that occurs upon microglia activation [10]. It was shown that activated microglia export apolipoprotein E and J involved in lipid transport [11]. Apolipoprotein E was linked to microglial activation and to increased neurotoxicity in several publications [12], [13]. Our enrichment-mapping analysis also revealed all components of tumor necrosis factor (TNF)-NF-kappa-B pathway as significant. IFN-gamma-dependent production of TNF-alpha mediated by STAT1 was previously reported [14]. Activation of NF-kappa-B suggests activation of apoptosis in microglia that was also documented previously [15]. Thus, TNF-alpha can also provide a negative feedback loop to slow-down the spread of microglia activated by IFN-gamma. One possible scenario is that IFN-gamma induction activates microglia migration towards the injury site and initiates TNF-alpha release to kill the damaged neurons, but also to initiate death of activated microglia in order to stop the spread of inflammation beyond the site of injury.

**Figure 5:**
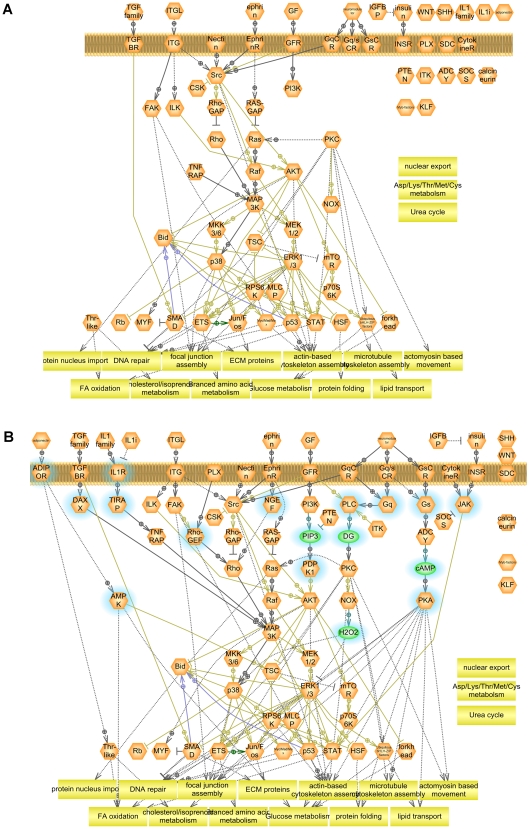
Projection of groups involved in TGF-beta response in lamina cribrosa cells onto Atlas of Signaling. (A) The protein groups found by projection of the results of enrichment analysis of GEO dataset GDS1313 were connected using existing relations in the Atlas of Signaling. (B) Significant groups not connected by direct relations from the Atlas were linked to the core network by adding minimal number of secondary messengers (green ovals) and functional classes not significant according to enrichment results (highlighted in blue). Group shapes are as described in legend to Figure 2.

Next, we analyzed the experiment GDS1313 which profiles the response of the glial fibrillary acidic protein (GFAP)-negative lamina cribrosa (LC) cells to a 24-hour exposure to Transforming Growth Factor beta (TGF-beta) 1 at 10 ng/ml concentration. TGF beta is a multifunctional protein that controls proliferation, differentiation, and other functions in many cell types. Application of the Transforming Growth Factors to normal rat kidney fibroblasts induces proliferation in cultured cells that results in overgrow that is no longer subjected to the normal cell-contact inhibition. The GFAP-negative LC cells are used as a model for primary open-angle glaucoma (POAG) when TGF-beta level is elevated in human LC tissue. We have performed a similar analytical workflow: differential gene expression followed by enrichment analysis against “expression sub-network” gene sets followed by mapping of significant sub-network seeds to the Atlas of Signaling. The results of the analysis are shown on Figure 5. As expected, the classical TGFbeta/SMAD pathway is identified as significant along with the extracellular matrix proteins that are directly regulated by SMADs. Involvement of extracellular matrix (ECM) proteins identified by our analysis is in perfect agreement with the fact that pathological hallmarks of the glaucomatous optic nerve head include retinal ganglion cell axon loss and extracellular matrix (ECM) remodeling of the lamina cribrosa layer [16]. Interestingly, the results also suggest involvement of p38 MAPK, AKT and PKA pathways in the observed response which regulate ECM deposition, cytoskeleton and focal junction assembly, protein folding and several metabolic processes (Figure 5A). There is a handful of unconnected protein classes (upper right corner of the map), but they can be joined to the core network by a few secondary messengers or functional classes whose expression was not significantly affected by TGF-beta treatment (Figure 5B).

Finally, we have analyzed the experiment GDS1543 from NCBI GEO repository measuring the effect of tumor necrosis factor (TNF)-alpha on microvascular endothelial cells (HMEC) (5 h, 2 ng/ml TNF). TNF-alpha is a cytokine involved in systemic inflammation and is a member of a group of cytokines that stimulate the acute phase reaction. It is mainly secreted by macrophages and can induce cell death of certain tumor cell lines. It is a potent pyrogen causing fever by direct action or by stimulation of interleukin-1 secretion. Under certain conditions it can stimulate cell proliferation and induce cell differentiation.

**Figure 6:**
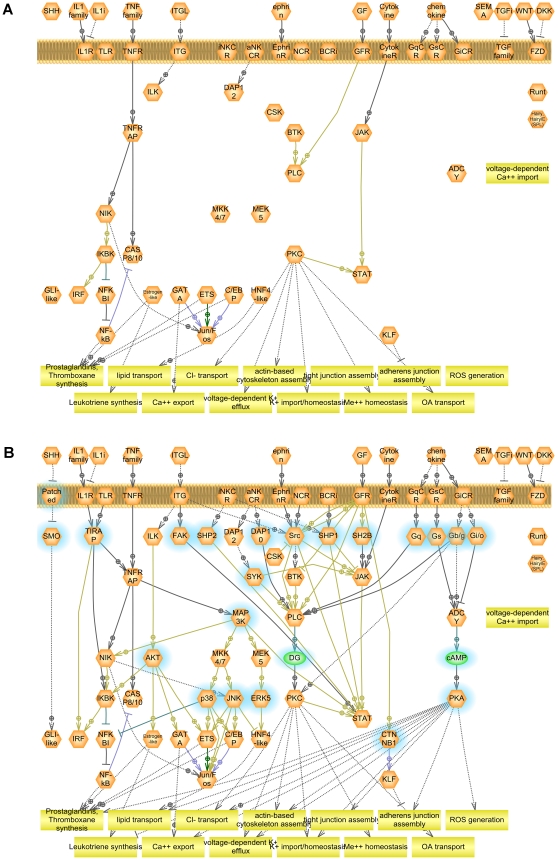
Projection of groups involved in TNF-alpha response in microvascular endothelial cells onto Atlas of Signaling. GEO dataset GDS1543 was analyzed as described in legend to Figure 5.

For the analysis, 2 samples (GSM50775 and GSM50773) were excluded from the experiment because they have shown an inconsistent clustering: they represent control and treatment samples which clustered to each other as opposed to clustering with other control and treatment samples. The pathway obtained by mapping significant sub-network seeds on the Atlas of Signaling is shown on Figure 6.

The universal feature of the TNF pathway conserved in evolution and among different human tissues is believed to be the activation of NF-kappa-B transcription factor [17]. Our analysis has revealed an activation of NF-kappa-B in HMEC cells. Nevertheless, TNF-alpha exhibits diverse effects on different tissues [18], [19]. The tissue specificity of TNF-alpha action is determined by the tissue-specific expression of TNF receptor-associated adaptors acting as scaffolds to associate different sets of downstream signaling molecules with TNF-receptor in different tissues, resulting in regulation of tissue-specific processes [20]. Gene expression analysis helps to select a particular set of such target processes. For instance, our analysis revealed large number of transport processes affected in HMEC cells by TNF-alpha treatment. This can be a specific feature of endothelial cells. On the other hand, membrane depolarization [21] and changes in cell volume due to deregulation of ion homeostasis [22] is now believed to play a pivotal role in early stages of apoptosis, which is the main process regulated by NF-kappa-B.

In conclusion, we have developed a novel approach for a systems-level interpretation of microarray expression data in the context of manually constructed “overview” pathway depicting the main cellular signaling channels. Using publicly available gene expression datasets, we have demonstrated that the developed approach can highlight the biologically plausible sub-networks of the global cellular signaling network. We believe that the developed approach can be used for general systems-level interpretation of differential expression profiling experiments.

## Supporting Information

File S1 Complete list of protein classes in Ontology.
(0.52 MB DOC)

File S2 Relations in the Atlas of Signaling.
(0.10 MB XLS)
