# Supplementary material for: Atlas of Signaling for Interpretation of Microarray Experiments
Source: PLoS One. 2010 Feb 17;5(2):e9256. doi: 10.1371/journal.pone.0009256 (PMC2822851; doi:10.1371/journal.pone.0009256)
Supplement: File S1 — Complete list of protein classes in Ontology. (0.52 MB DOC) [file pone.0009256.s001.doc]

**Biochemical processes (pathways).**

The set of biochemical pathways is designed to cover all major aspects of animal metabolism, based primarily on biochemical textbook and KEGG. Proteins were assigned to each group based on their EC number annotation in EBI Enzyme database. Table below shows list of biochemical pathways and number of proteins in each process. Total number of proteins assigned to biochemical processes: 1579

| **Process** | **Proteins** |
| --- | --- |
| Amino sugars synthesis | 15 |
| Aromatic aminoacid metabolism | 128 |
| Asp/Lys/Thr/Met/Cys metabolsm | 39 |
| Bile acid metabolism | 9 |
| Branced aminoacid metabolism | 46 |
| Cholesterol/isoprenoid metabolism | 28 |
| Fatty acid biosynthesis | 15 |
| Fatty acid oxidation | 45 |
| Folate and pterins biosynthesis | 15 |
| Glucose metabolism | 176 |
| Glut/Gln/Pro metabolsm | 32 |
| Glycan catabolism | 15 |
| Glycogen degradation | 22 |
| Glycogen synthesis | 9 |
| Glycosylation in Golgi | 119 |
| GPI anchor biosynthesis | 24 |
| Heme oxidation | 24 |
| His metabolism | 5 |
| Leukotriene synthesis | 35 |
| Lipid degradation | 40 |
| Lysosomal targeting/Mannose phosphorylation | 2 |
| Mannose biosynthesis | 9 |
| NAD biosynthesis | 5 |
| N-Glycan biosynthesis (ER) | 43 |
| NO biosyntheis | 6 |
| Pentose-phosphate shunt | 22 |
| Phospholipid biosynthesis | 90 |
| Phospholipid scrambling | 5 |
| Prostaglandins, Thromboxane synthesis | 12 |
| Purine metabolism | 96 |
| Pyrimidine metabolism | 59 |
| Respiratory chain | 140 |
| ROS catabolism | 10 |
| ROS generation | 11 |
| Ser/Gly metabolism | 44 |
| Steroid metabolism | 60 |
| Tetrapyrroles biosynthesis | 17 |
| Triacylglycerols biosynthesis | 14 |
| Triacylglycerols degradation | 14 |
| Tricarboxylic acid cycle | 39 |
| Urea cycle | 40 |

**Transport processes.**

Set of transport processes are designed to cover major functional fluxes of metabolites and ions. The “identity” of transport process is determined by combination of:

- Metabolite or ion
- transport direction relative to cytoplasm
- compartmentalization (other than cytoplasm)
- active transport mechanism: ATP dependent, ATP independent or voltage dependant
- co- or anti- transported compounds

Table below shows list of transport processes and number of proteins for each class meta-class. Total number of proteins assigned to transport processes: 1056

| **Process** | **Proteins** |
| --- | --- |
| AA import | 29 |
| Ca++ ER import | 5 |
| Ca++ export | 13 |
| Cholesterol export | 5 |
| Choline import | 3 |
| Cl- transport | 77 |
| Endolysosomal AA export | 2 |
| ER Ca++ release | 11 |
| Fatty acids import | 5 |
| Glucose import | 18 |
| H+ homeostasis/export | 11 |
| HCO(3)(-) transport | 16 |
| K+ import/homeostasis | 48 |
| Lipid export | 11 |
| Lipid transport | 55 |
| Lysosomal H+ import | 26 |
| Me++ homeostasis | 52 |
| Mitochondrial CytC release | 2 |
| Mono-/di-carboxylate import | 15 |
| Na+ homeostasis/export | 21 |
| Na+ influx co-transport | 96 |
| Neurotransmitter loading | 7 |
| Neurotransmitter uptake | 25 |
| Non-voltage Ca++ import | 34 |
| Non-voltage K+ efflux | 47 |
| Non-voltage Na+ influx | 19 |
| Nucleoside transport | 6 |
| OA transport | 45 |
| Oligopeptide import | 4 |
| Other transport proteins | 170 |
| Phosphate import | 9 |
| Phospholipid flipping | 14 |
| Sulfate import | 9 |
| Thyroid hormone import | 4 |
| Urea export | 2 |
| Vitamin import | 11 |
| Voltage-dependent Ca++ import | 30 |
| Voltage-dependent K+ efflux | 47 |
| Voltage-dependent K+ influx | 8 |
| Voltage-gated Na+ influx | 14 |
| Water transport | 10 |
| Xenobiotic clearance | 20 |

**Structural processes**

Structural processes were designed to represent the major tissue independent ubiquitous processes occurring in a cell. The proteins were assigned to these processes only if they directly execute the process or are direct specific regulators of the process.

Table below shows list of processes and number of proteins in each. Total number of proteins assigned to structural processes: 3821

| **Process** | **Proteins** |
| --- | --- |
| Actin-based cytoskeleton assembly | 210 |
| Actomyosin based movement | 74 |
| Adherens junction assembly | 47 |
| Centriole duplication | 37 |
| Centrosome separation | 36 |
| Chromatin remodeling | 223 |
| Chromosome condensation | 7 |
| Cleavage of lamina in apoptosis | 2 |
| Co-translational ER protein import | 22 |
| desmosome assembly | 18 |
| DNA degradation | 4 |
| DNA recombination | 28 |
| DNA repair | 109 |
| DNA replication | 64 |
| ECM degradation | 14 |
| ECM/Cell adhesion proteins | 156 |
| Endocytosis | 72 |
| Endosomal recycling | 49 |
| Endosome - lysosome transport | 98 |
| ER to Golgi transport | 49 |
| Extracellular matrix polymerization | 97 |
| Focal junction assembly | 37 |
| Gap junction assembly | 30 |
| Golgi to endosome transport | 37 |
| Hemidesmosome assembly | 15 |
| Intermediate filament polymerization | 134 |
| Intra-Golgi transport | 22 |
| Kinetochore assembly | 49 |
| Lysosomal lumen acidification | 8 |
| Microtubule cytoskeleton assembly | 153 |
| Microtubule sliding | 167 |
| Mitochondrial protein transport | 23 |
| Mitochondrial transcription | 1 |
| Mitochondrion fission | 3 |
| Mitochondrion fusion | 1 |
| mRNA processing | 196 |
| Nuclear export | 75 |
| Nuclear pore organization and biogenesis | 26 |
| Nucleolus organization and biogenesis | 115 |
| Peroxisome division | 3 |
| Peroxisome protein import | 12 |
| Polymerase I transcription | 39 |
| Polymerase II transcription | 255 |
| Polymerase III transcription | 63 |
| Presentation of endogenous peptide antigen | 10 |
| Protein folding | 88 |
| Protein nucleus import | 53 |
| Retrograde endosome-Golgi transport | 39 |
| Retrograde Golgi-ER transport | 20 |
| Retrograde protein transport, ER to cytosol | 9 |
| RNA degradation | 10 |
| RNA gene silencing | 14 |
| rRNA processing | 16 |
| Sister chromatid cohesion | 11 |
| Spindle assembly | 62 |
| Telomere maintenance | 14 |
| Tight junction assembly | 62 |
| Transcytosis | 12 |
| Translation | 228 |
| tRNA processing | 15 |
| Ubiquitin-dependent protein degradation | 166 |
| Vesicular secretory pathway | 112 |

**Ligands**

Groups of extracellular signaling proteins acting through membrane receptors. Total number of ligand proteins: 444

| **Family** | **Proteins** |
| --- | --- |
| Adiponectin | 2 |
| agouti | 2 |
| B7 | 2 |
| B7coi | 3 |
| B7cos | 2 |
| Chemokines | 47 |
| Chromogranin | 2 |
| Coagulation factor | 11 |
| Cytokine | 63 |
| DKK | 6 |
| DLL | 3 |
| DLLi | 1 |
| Ephrin | 7 |
| GDNF | 4 |
| GF | 54 |
| GUC activator | 5 |
| HAMP | 1 |
| IGFBP | 7 |
| IL1 family | 6 |
| IL1i | 5 |
| INS | 7 |
| ITGL | 14 |
| LH | 7 |
| MDK | 1 |
| netrin | 5 |
| Neuromodulator | 62 |
| NGF family | 4 |
| Nogo | 3 |
| NXPH | 4 |
| PLA | 2 |
| PTN | 1 |
| RETN | 2 |
| SELLG | 1 |
| SEMA | 21 |
| SHH | 3 |
| SLIT | 3 |
| SLRP | 1 |
| STC | 1 |
| TGF family | 25 |
| TGFi | 10 |
| TNF family | 18 |

**Receptors**

The set of *Receptor* groups are designed based on their ligand specificity; signaling pathway downstream of a receptor; tissue specificity and protein sequence similarity to other members of the group. Total number of receptor proteins: 929

| **family** | **Proteins** |
| --- | --- |
| ADIPOR | 2 |
| ALK | 1 |
| aNKCR | 16 |
| BCR | 6 |
| BCRi | 2 |
| CD3Z | 2 |
| CD44 | 1 |
| Cytokine Receptors | 46 |
| DCC | 1 |
| DDR | 2 |
| Ephrin R | 13 |
| FCeR | 2 |
| Folate receptor | 3 |
| FZD | 13 |
| GCR | 9 |
| GDNFR | 5 |
| GFR | 25 |
| GiCR | 79 |
| GPCR | 138 |
| Gq/12CR | 2 |
| Gq/iCR | 19 |
| Gq/sCR | 8 |
| GqCR | 70 |
| GsCR | 39 |
| IL1R | 5 |
| IL1Rd | 1 |
| IL1RL1 | 1 |
| iNKCR | 13 |
| INSR | 5 |
| ITG | 28 |
| Low-density lipoprotein (LDL) receptor | 10 |
| LRR-Ig receptor | 4 |
| NCR | 6 |
| Nectin | 5 |
| NEO | 3 |
| Neurexin | 4 |
| NGFR | 3 |
| NKCR | 1 |
| NogoR | 8 |
| Notch | 4 |
| NRP | 2 |
| NTRK | 3 |
| Patched | 2 |
| PLX | 8 |
| PTPRZ | 1 |
| RAMP | 3 |
| ROBO | 3 |
| ROBOi | 1 |
| ROR | 2 |
| RPTP | 14 |
| Scavenger receptor | 26 |
| SDC | 4 |
| Selectin | 3 |
| TCR | 123 |
| TCRcoi | 3 |
| TCRcos | 2 |
| Tetraspanin | 31 |
| TGFBR | 16 |
| TIGIRR | 2 |
| TLR | 12 |
| TNFR | 30 |
| Transferrin receptor | 3 |
| UNC | 4 |
| uPAR | 1 |

**Signaling proteins**

The groups (families) of signaling proteins are designed based on their sequence similarity and pathway specificity. Total number of signaling signaling proteins: 744

| **family** | **Proteins** |
| --- | --- |
| Calcineurin | 6 |
| CDC25A/B/C | 3 |
| DUSP | 13 |
| INSR phosphatase | 2 |
| MKP | 3 |
| MLCP | 5 |
| PP2A | 7 |
| PP2CA | 1 |
| PP5/2CB | 2 |
| PTEN | 1 |
| SHP1 | 1 |
| SHP2 | 1 |
| ABL | 2 |
| AKT | 3 |
| AMPK | 7 |
| ATM/ATR | 2 |
| CAMK | 12 |
| CAMKK | 2 |
| CASK | 1 |
| CDK1 | 1 |
| CDK2 | 1 |
| CDK4/6 | 2 |
| CDK7 | 1 |
| CHEK | 2 |
| EEF2K | 1 |
| ERK1/3 | 3 |
| ERK5 | 1 |
| FAK | 2 |
| GSK3 | 2 |
| HIPK | 4 |
| IKBK | 5 |
| ILK | 1 |
| JAK | 4 |
| JNK | 3 |
| LIMK | 2 |
| MAP3K | 20 |
| MAP4K | 4 |
| MAPKAPK | 3 |
| MEK1/2 | 2 |
| MEK5 | 1 |
| MKK3/6 | 2 |
| MKK4/7 | 2 |
| MLCK | 3 |
| MNK1/2 | 2 |
| NIK | 1 |
| NLK | 1 |
| p38 | 4 |
| p70S6K | 2 |
| PAK | 3 |
| PDPK1 | 2 |
| PHK | 5 |
| PKA | 9 |
| PKC | 11 |
| PKG | 2 |
| PRKD | 3 |
| Raf | 4 |
| ROCK1/2 | 2 |
| RPS6K | 5 |
| SYK | 2 |
| Wee/Myt1 | 2 |
| ADCY | 9 |
| AIF | 1 |
| APAF1 | 1 |
| APC/C | 11 |
| ARF | 7 |
| AXIN | 2 |
| Bcl | 6 |
| Bid | 6 |
| BTK | 1 |
| CALM | 12 |
| CASP12 | 1 |
| CASP3/6/7 | 3 |
| CASP8/10 | 2 |
| CASP9 | 1 |
| CBL | 3 |
| CD38 | 2 |
| CDKN | 7 |
| Crk | 2 |
| CSK | 1 |
| CTNNB1 | 1 |
| CycA | 2 |
| CycB | 3 |
| CycD | 3 |
| CycE | 2 |
| CycH | 1 |
| CytC | 1 |
| DAAM | 2 |
| DAP10 | 1 |
| DAP12 | 1 |
| DAXX | 1 |
| DIABLO | 1 |
| DOCK | 5 |
| Dvl | 4 |
| FLIP | 1 |
| FRAT | 2 |
| G proteins a12/13 | 2 |
| GADD45 | 3 |
| G proteins b/g | 18 |
| G proteins i/o | 4 |
| GIT1 | 1 |
| G proteins q | 4 |
| Grb | 3 |
| GRK | 7 |
| G proteins s | 2 |
| G proteins t | 3 |
| G proteins z | 1 |
| IAP | 7 |
| IQGAP | 3 |
| IRS | 3 |
| ITK | 2 |
| ITPK | 2 |
| ITPR | 4 |
| LAT | 1 |
| mDia | 3 |
| MDM2/4 | 2 |
| mTOR | 2 |
| NCS1 | 1 |
| NFKBI | 4 |
| NGEF | 1 |
| NOS | 5 |
| NOX | 11 |
| p130CAS | 1 |
| PDE1 | 3 |
| PDE6 | 4 |
| PI3K | 13 |
| PLA2 | 15 |
| PLC | 19 |
| PSC | 2 |
| Rac/CDC42 | 4 |
| Rac/Cdc-GAP | 4 |
| Rac/Cdc-GEF | 2 |
| RAP | 5 |
| RAP-GAP | 2 |
| RAP-GEF | 7 |
| Ras | 7 |
| RAS-GAP | 8 |
| RAS-GRF | 9 |
| RGS | 25 |
| Rho | 3 |
| Rho-GAP | 4 |
| Rho-GEF | 5 |
| RYR | 3 |
| SCF | 4 |
| sGC | 4 |
| SGK | 1 |
| SH2B | 1 |
| Shc | 5 |
| SLP76 | 1 |
| SOCS | 6 |
| SOS | 2 |
| Src | 5 |
| TIRAP | 9 |
| TNFRAP | 7 |
| VAV | 5 |

**Transcription factors**

Proteins were classified into different Transcription factors groups based on their sequence similarity similarities. Total number of transcription factors: 1841.

| **Family** | **Proteins** |
| --- | --- |
| Achaete-Scute | 4 |
| AP-2 | 5 |
| Atonal | 14 |
| bZIP/PAR | 4 |
| C/EBP | 6 |
| CCAAT factors | 3 |
| CNBP | 1 |
| CREB | 9 |
| CRE-BP/ATF | 6 |
| Csd/Coldshock | 3 |
| DP | 3 |
| E2F | 8 |
| Egr/Krox | 4 |
| Estrogen-like | 9 |
| Ets | 26 |
| Forkhead | 41 |
| Fushi tarazu-F1-like | 2 |
| GATA | 6 |
| Germ cell nuclear factor | 1 |
| GLI-like | 7 |
| Grainyhead | 6 |
| Hairy, Hairy/E(SPL) | 11 |
| HEN | 2 |
| HIVEP | 3 |
| HLH domain only | 4 |
| HMG2-related | 1 |
| HMGI(Y) | 2 |
| HNF4-like | 12 |
| Homeo | 8 |
| Homeo/AbdB | 17 |
| Homeo/Antp | 27 |
| Homeo/Cad | 3 |
| Homeo/Cut | 2 |
| Homeo/DII | 6 |
| Homeo/Ems | 4 |
| Homeo/En | 2 |
| Homeo/Eve | 6 |
| Homeo/HK2 | 16 |
| Homeo/HLX | 4 |
| Homeo/HNF1 | 2 |
| Homeo/LIM | 15 |
| Homeo/Msh | 2 |
| Homeo/PBC | 4 |
| Homeo/POU | 14 |
| Homeo/Prd | 21 |
| Homeo/XANF | 1 |
| Homeo/ZF | 2 |
| HSF | 3 |
| ID | 4 |
| IRF | 9 |
| iSMAD | 2 |
| Jun/Fos | 12 |
| KLF | 371 |
| Knirps/DAX 1-like | 2 |
| Maf | 7 |
| MEF-2 | 4 |
| Mesp | 3 |
| Myb-factors | 4 |
| Myc/Mad/Max | 12 |
| MYF | 2 |
| Nerve growth factor IB-like | 3 |
| NF-1 | 4 |
| NF-AT | 5 |
| NF-E2 | 6 |
| NF-kB | 5 |
| Other NR | 1 |
| p53 | 1 |
| Paired box | 9 |
| Pancreatic factors | 2 |
| PAS domain | 18 |
| PDX | 1 |
| Rb | 3 |
| RBPJ | 2 |
| RF-X | 5 |
| Runt | 3 |
| SMAD | 8 |
| SOX | 20 |
| SP | 10 |
| SRF | 1 |
| STAT | 7 |
| TAL | 3 |
| TCF-1 | 4 |
| TCF3/4/12 | 3 |
| TEA | 4 |
| Teashirt | 3 |
| Thr-like | 19 |
| Trithorax | 5 |
| Twist-like | 7 |
| Ubiquitous bHLH-ZIP factors | 9 |
| UBTF | 1 |
| ZIP only | 1 |
